# Study protocol of the METAPANC trial - intensified treatment in patients with local operable but oligometastatic pancreatic cancer - multimodal surgical treatment versus chemotherapy alone: a randomized controlled trial

**DOI:** 10.1186/s12885-025-13573-7

**Published:** 2025-02-06

**Authors:** Michael Ghadimi, Uwe Pelzer, Marc G. Besselink, Jens Siveke, Ralph Telgmann, Rickmer Braren, Hanneke Wilmink, Marie Crede, Alexander Koenig, Ute Koenig, Sven Thorsten Liffers, Kai Antweiler, Bas Uijterwijk, Hanna Seppanen, Arno Nordin, Pauli Puolakkainen, Olav F. Dajani, Knut Jørgen Labori, Mia Johansson, Svein Olav Bratlie, Tim Friede, Peter Jo

**Affiliations:** 1 Department of General, Visceral and Pediatric Surgery, University Medical Center Goettingen, Goettingen, Germany; 2 Division of Oncology and Hematology, Charité Campus Mitte, Charité - Universitätsmedizin Berlin, Freie Universität Berlin, Humboldt-Universität zu Berlin, Berlin Institute of Health, Berlin, Germany; 3 Department of Surgery, Amsterdam UMC, University of Amsterdam, Amsterdam, The Netherlands; 4 University Hospital Essen, West German Cancer Center, Essen, Germany; 5 Clinical Trials Unit, University Medical Center Goettingen, Goettingen, Germany; 6 Institute of Diagnostic and Interventional Radiology, Technical University of Munich, Munich, Germany; 7 Department of Medical Oncology, Amsterdam UMC, University of Amsterdam, Amsterdam, The Netherlands; 8 Department of Gastroenterology and Gastrointestinal Oncology, University Medical Center Goettingen, Goettingen, Germany; 9 Department of Medical Statistics, University Medical Center Goettingen, Goettingen, Germany; 10 Department of Gastrointestinal Surgery, University of Helsinki and Helsinki University Hospital, Helsinki, Finland; 11 Department of Abdominal Surgery, University of Helsinki and Helsinki University Hospital, Helsinki, Finland; 12 Department of Oncology, Oslo University Hospital, Oslo, Norway; 13 Department of Hepato Pancreato Biliary Surgery, Oslo University Hospital, Oslo, Norway; 14 Department of Oncology, Sahlgrenska University Hospital, Gothenburg, Sweden; 15 Department of Surgery, Sahlgrenska University Hospital, Gothenburg, Sweden; 16 Department of General, Visceral and Pediatric Surgery, University Medical Center Göttingen, Robert-Koch-Straße 40, 37077 Goettingen, Germany

**Keywords:** Oligometastasis, Pancreatic ductal carcinoma (PDAC), Oligometastatic pancreatic cancer, Liver metastasis, Multimodal treatment, Chemotherapy, Pancreatic surgery, Clinical trials

## Abstract

**Background:**

Based on current guidelines, surgical treatment of hepatic oligometastases in patients with pancreatic ductal adenocarcinoma (PDAC) is not primarily recommended. Systematic chemotherapy is the therapy of choice for these patients. The relevance of subsequent surgical resection after chemotherapy remains unclear. This multicentre, randomized, controlled phase III trial is planned to evaluate whether resection of the primary tumor and liver metastases can improve overall survival in patients with PDAC with hepatic oligometastases in a multimodal treatment setting.

**Methods:**

After an induction therapy with eight cyles of mFOLFIRINOX and a response assessment after four and eight cycles, patients will be randomized to either Arm 1 (perioperative mFOFIRINOX plus resection of the primary tumor with resection or ablation of all hepatic metastases) or Arm 2 (continuation of 4 cycles of the standard-of-care mFOLFIRINOX chemotherapy). This clinical trial will focus on a well-defined patient group with metastatic disease limited to the liver as the target organ, with a maximum of three metastases.

**Discussion:**

METAPANC is the first international, randomized, controlled, open-label, multicentre, phase III clinical trial for curative intended surgical therapy of oligometastatic pancreatic cancer in Europe and America. The multimodal surgical treatment of patients with oligometastatic pancreatic cancer could significantly extend the overall survival of this patient group. A possible recommendation of this multimodal treatment regimen outside of clinical trials requires data from randomized controlled trials first. To identify patient subgroups that might benefit from multimodal surgical therapy, additional information on tumor genetics could supplement valid parameters.

**Trial registration:**

EU Clinical Trials No. 2023-503558-10-00.

**Supplementary Information:**

The online version contains supplementary material available at 10.1186/s12885-025-13573-7.

## Background

Pancreatic ductal adenocarcinoma (PDAC) has a poor overall survival rate of 8% due to early metastasis and therapy resistance. The successful introduction of intensified chemotherapeutic regimens, such as FOLFIRINOX and gemcitabine/nab-paclitaxel, has led to a significant, though still moderate, improvement in response rates, disease control, and overall survival. Current standards recommend systemic chemotherapy but no surgical approach in metastatic (stage IV) PDAC. FOLFIRINOX has the highest rates of response and overall survival and is a current standard-of-care chemotherapy regimen in patients with metastatic PDAC and good performance status [1]. Given the high rates of recurrence, and without available biomarkers for potential target populations that may benefit from extensive and complex surgical interventions, synchronous or metachronous metastasectomy is not recommended and only rarely performed in individual cases.

However, pancreatic resections, including pancreaticoduodenectomy, distal pancreatectomy, and total pancreatectomy, resection of metastases have reached a high-quality level with considerably low morbidity and < 5% mortality in high-volume centres. Several case studies have reported favorable outcomes for selected patients with PDAC undergoing resection of metastases [2, 3] and highly selected patients with oligometastatic PDAC and good response to intensified chemotherapy may potentially benefit from multimodal approaches. Recently, the Chinese Study Group for Pancreatic Cancer (CSPAC) reported on a multicentre, randomized, controlled phase III trial [4], using a similar definition as used in the here proposed METAPANC trial design. In CSPAC-1, various first-line chemotherapeutic regimen are used, and time of treatment and randomization prior to the surgery or control arm is variable. CSPAC-1 aims is to establish a treatment strategy to select patients who might benefit from simultaneous resection of primary pancreatic cancer and liver metastatic sites. The trial is expected to run for at least 7 years. METAPANC uses a similar yet distinct study protocol and is the first international, randomized, controlled, open-label, multicentre, phase III clinical trial for curative intended surgical therapy of oligometastatic pancreatic cancer in Western Europe.

## Objectives and endpoints

### Objectives

**Fig. 1:**
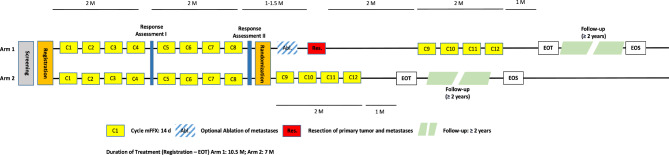
Flow chart of the entire study including the optional percutaneous interventional ablation of metastases in Arm 1. In these cases the liver ablation of selected metastatic lesion(s) can be planned 1–3 weeks after the last mFOLFIRINOX cycle, but within six weeks between the last cycle of chemotherapy and surgery. Alternatively, the ablation can be conducted intraoperatively

The primary objective of the METAPANC trial is to evaluate the efficacy of neoadjuvant multimodal chemotherapy followed by complete tumor and metastases resection in patients with hepatic oligometastatic PDAC. The principal research question is whether overall survival in patients with oligometastatic pancreatic cancer is superior in patients treated with perioperative modified FOLFIRINOX (mFOLFIRINOX) followed by complete surgical resection compared to standard-of-care mFOLFIRINOX first-line chemotherapy alone. In a prospective randomized trial design, we aim to evaluate survival and quality of life after multimodal therapy with perioperative mFOLFIRINOX chemotherapy (two times 4 cycles prior to resection of the primary tumor and resection / ablation of the metastases), followed by 4 cycles of adjuvant mFOLFIRINOX chemotherapy (total of 12 cycles) vs. mFOLFIRINOX without surgery / ablation (total of 12 cycles) as standard of care first-line treatment (Fig. 1). All patients will be followed-up for at least 2 years. Secondary objectives are to determine the safety of the treatment concept and health-related quality of life. Hereby we explore procedure-related complications and mortality. Furthermore, we seek to evaluate biomarkers and imaging data for better identification of a potential target population which may benefit from intensified multimodal treatment strategies.

### Endpoints

The primary endpoint is overall survival (OS) as the time from randomization to death of any cause. Secondary endpoints include progression-free survival (PFS), quality of life (EORTC QLQ-C30, PAN-26, Q-TWIST) and translational endpoints. PFS is defined as the time from randomization to death or progression whatever occurs first. During the induction phase of the study, response will be assessed according to RECIST v1.1. Progression is based on radiological assessment according to RECIST v1.1.

For translational research purposes, tissue from the initial diagnostic biopsy and the resection specimen will be collected, if possible from both tumor tissue from the primary tumor and from metastases (this should be feasible at least for patients that were surgically explored). Aims are to identify predisposing factors for metastasis, response to mFOLFIRINOX treatment and adaptive molecular changes under chemotherapy. Characterization will address the molecular subtype and clonal evolution to better understand mechanisms of metastasis and to identify chemotherapy response / resistance patterns.

Blood samples are taken before each chemotherapeutic cycle, before surgical resection and during maintenance therapy and surveillance. Analysis will focus on circulating tumor DNA, analysis for monitoring of mutational patterns of pancreatic cancer during cancer therapy (prognostic for disease survival, predictive for primary or secondary drug resistance) in comparison to currently used tumor markers (CA19- 9) using e.g. a multiplex amplicon-based sequencing approach (Table 1).

**Table 1:** Objectives and endpoints of the METAPANC trial

**1. Objectives**	
1.1. Primary Objectives	• To assess the efficacy of neoadjuvant multimodal chemotherapy followed by complete tumor and metastases resection in patients with hepatic oligometastatic PDAC
1.2. Secondary Objectives	• To determine the efficacy and safety of the treatment concept • To determine health-related quality of life (HR-QoL)
1.3. Other Exploratory Objectives	• To evaluate biomarkers and imaging data for better identification of a potential target population
**2. Endpoints**	
2.1. Primary Endpoint	• Overall survival as the time from randomization to death of any cause
2.2. Secondary Endpoints	• *Efficacy*: Progression-free survival • *Safety*: grading of the severity/intensity of an adverse event: the National Cancer Institute Common Toxicity Criteria for Adverse Events (NCI CTCAE) version 5.0 • *Health-Related Quality of Life*: EORTC QLQ-C30, PAN-26, Q-TWIST • *Exploratory Endpoints*: Translational endpoints

## Methods/study design

METAPANC is an international, randomized, controlled, open-label, multicentre, phase III clinical trial. This trial is composed of two arms, and patients will be randomized to either Arm 1 (perioperative mFOLFIRINOX plus resection of all cancer lesions) or Arm 2 (standard-of-care mFOLFIRINOX chemotherapy). Prior to randomization, all patients will undergo 8 cycles of mFOLFIRINOX induction therapy with response assessment after the fourth and eighth cycles. Patients in Arm 1 will be treated for approximately 12 months (induction chemotherapy with mFOLFIRINOX) followed by surgery and adjuvant of 4 cycles of mFOLFIRINOX-chemotherapy. Patients in Arm 2 will be treated for approximately 6 months with 12 cycles of mFOLFIRINOX-chemotherapy. Follow-up visits will be performed for all patients for a minimum of two years.

### Trial population

Patients with hepatic oligometastatic pancreatic ductal adenocarcinoma with a maximum of 3 liver metastases who meet the inclusion criteria and do not meet the exclusion criteria will be eligible to participate in this clinical trial.

### Inclusion criteria

Patients eligible for inclusion in this trial must meet all the following criteria:

1. Age ≥ 18 years and ≤ 80 years.
2. histologically or cytologically confirmed metastatic adenocarcinoma of the pancreas.
3. medical and technical operability of the primary tumor.
4. limited synchronous liver metastatic status (≤ 3 resectable/ ablatively treatable liver metastases).
  OR.
  limited metachronous liver metastatic status (≤ 3 resectable/ ablatively treatable liver metastases), but must have completed adjuvant chemotherapy at least 6 months before start of study treatment.

5. previous neo-/adjuvant anti-cancer therapy for non-metastatic PDAC with last dose administered ≥ 6 months before the start of study treatment are allowed.
6. adequate hematological (WBC ≥ 3000/µl, platelets ≥ 100.000/µl, hemoglobin ≥ 8 g/dl), hepatic (bilirubin ≤ 2.5 x mg/dl) and renal function (creatinine clearance > 50 ml/min) parameters.
7. ECOG performance status ≤ 1.
8. written informed consent obtained according to international guidelines and local laws.
9. measurable disease according to RECIST v1.1. prior to induction therapy.

### Exclusion criteria

Patients eligible for this trial must not meet any of the following criteria:

1. Unresectable pancreatic cancer.
2. Prior chemotherapy within 6 months or prior radiation therapy within 28 days (e.g. in adjuvant settings).
3. Exception for previous systemic anti-cancer treatment for metastatic PDAC: Patients with need of immediate treatment (high tumour load, symptoms) may have received one cycle of FOLFIRINOX or modified FOLFIRINOX prior to study entry (Cycle 0) and may be enrolled.
4. Concurrent malignancy other than the disease under investigation with exception of malignancy that was treated curatively and has not recurred within 2 years prior to the date of screening. Fully resected basal or squamous cell skin cancers and any carcinoma in situ are eligible.
5. Patients with either peritoneal carcinomatosis or > 3 liver metastases or extrahepatic metastasis)
6. Known hypersensitivity to the active substances or any of the excipients.
7. Impaired cardiac function or clinically significant cardio-vascular disease, such as:
  - Congestive heart failure requiring treatment (NYHA grade > 2), or clinically significant arrhythmia (including uncontrolled atrial flutter/fibrillation).
  - Acute myocardial infarction, unstable angina pectoris, coronary stenting, or bypass surgery < 3 months prior to study entry.
8. Simultaneous participation in other interventional trials which could interfere with this trial; simultaneous participation in registries and diagnostic trials is allowed.
9. Inability to understand the study and/or comply with the protocol procedures.
10. Subject pregnant or breast feeding, or planning to become pregnant within 6 months.
11. Subject (male or female) is not willing to use highly effective methods of contraception (per institutional standard) during treatment and for 6 months (male or female) after the end of treatment (adequate: oral contraceptives, intrauterine device or barrier method in conjunction with spermicidal jelly).
12. Psychological, familial, sociological or geographical condition potentially hampering compliance with the study protocol and follow-up schedule. These conditions should be discussed with the patient prior to registration in the trial.

### Trial design

Patients with locally resectable but oligometastatic PDAC will be screened for study enrollment (approximately *n* = 400) according to the inclusion and exclusion criteria. If enrolled and registered for the study, patients will receive 2 × 4 cycles mFOLFIRINOX with response assessment after 4 and 8 cycles. Patients with progressive disease (PD) will be excluded from the trial. 272 patients with complete response (CR), partial response (PR) or stable disease (SD) according to RECIST v1.1 will be randomized (1:1) into Arm 1 (experimental arm) or Arm 2 (control arm).

In the experimental arm patients will receive at least eight cycles of mFOLFIRINOX followed by resection of the primary tumor and metastatic lesions, preferably in one procedure. Local ablation of liver metastases is allowed. After resection, patients receive adjuvant chemotherapy of 4 cycles of mFOLFIRINOX followed by surveillance / follow-up.

**Fig. 2:**
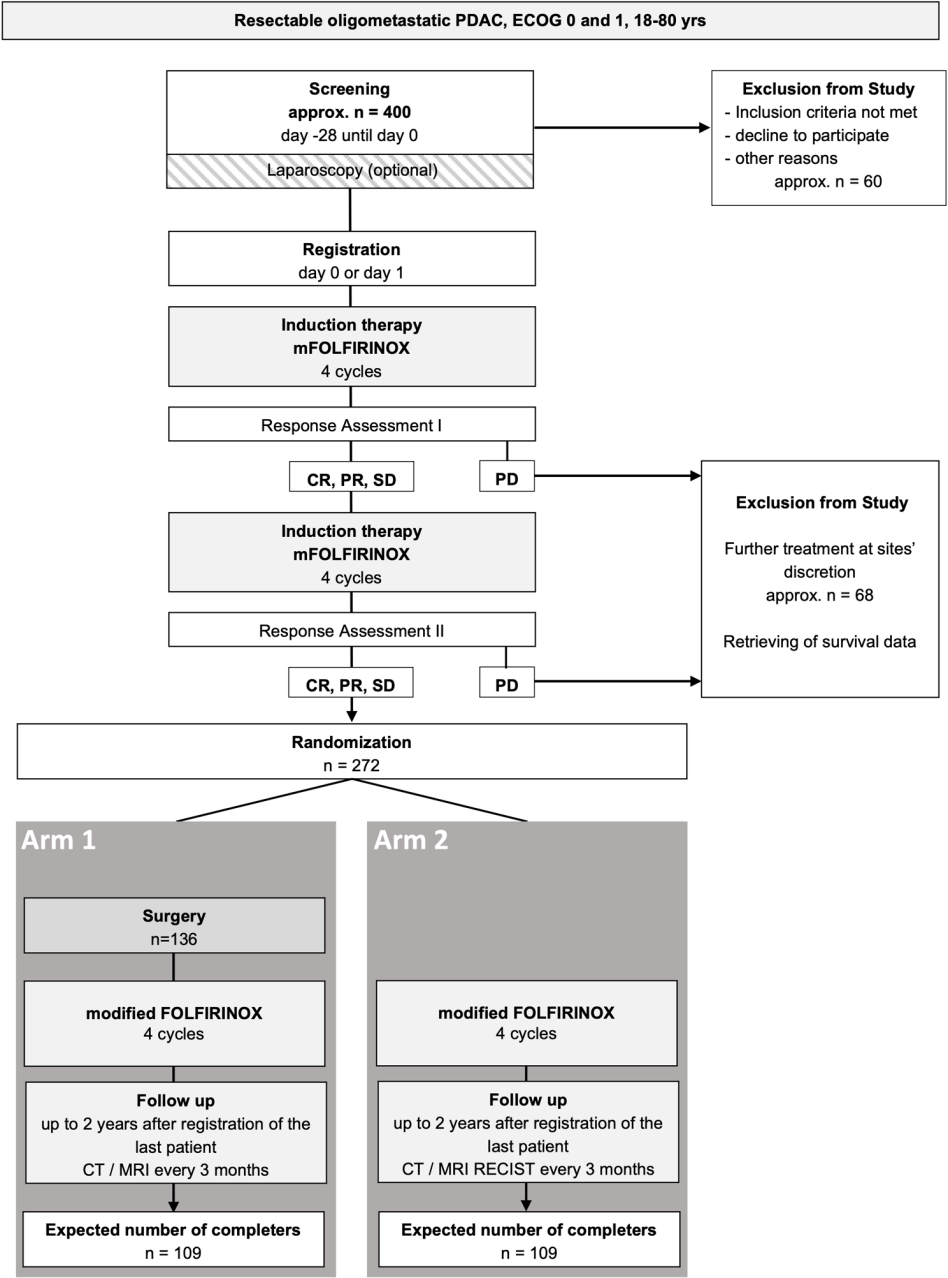
Flow chart of the METAPANC trial

In the control arm patients will receive at least 8 cycles of mFOLFIRINOX followed by adjuvant chemotherapy (mFOLFIRINOX for 4 cycles) followed by surveillance / follow-up. All Patients will undergo a follow up of 2 years. Figure 2 shows the study design as flow chart.

### Tumor imaging/laparoscopy

CT or MRI of abdomen with intravenous contrast agents according to specific protocols for imaging of the pancreas and local institutional practice as a standard-of-care procedure. CT/MRI may be used for imaging of the abdomen, Chest imaging by CT for detection of lung metastases. In patients, who have not been surgically explored before application of mFOLFIRNOX chemotherapy, a diagnostic laparoscopy is optional but recommended under certain conditions. The aim of the diagnostic laparoscopy is to exclude patients with peritoneal carcinomatosis or more than three visible liver metastases. In general, a diagnostic laparoscopy should be performed in cases of resectable pancreatic cancer under the following conditions: when imaging shows a large tumor (> 3 cm), or if ascites is present, or if there is an elevated tumor marker value of CA 19 − 9 > 500 U/ml, without cholestasis. In these situations, there is suspicion of occult organ metastasis (liver metastasis and/or peritoneal carcinomatosis). Intraoperative ultrasound, if available, of the liver during the diagnostic laparoscopy procedure is optional. MRI liver assessment at Response Assessment II is mandatory prior to randomization.

### Treatment regimen

Modified FOLFIRINOX (mFOLFIRNOX) is a combination of systemic chemotherapy agents. It consists of:

- Oxaliplatin at a dose of 85 mg/m2 given as a 2-hour intravenous infusion.
- Leucovorin at a dose of 400 mg/m2 given as a 30 min to 2-hour intravenous infusion.
- Irinotecan at a dose of 150 mg/m2 given as a 90-minute intravenous infusion.
- 5-FU at 2400 mg/m2 administrated as an intravenous infusion of over a 46-hour period.

Drugs will be used in its commercially available form and will be provided by the local pharmacies, all brands allowed and identified by the ACT code generic versions of oxaliplatin, irinotecan, leucovorin, and 5-FU if commercially available can be used according to local practice and local regulation. Preparation, doses and administration of the mFOLFIRINOX regimen will follow the institutional practice of the study site and instructions given in the local SmPCs. Regimen will be repeated every two weeks. Primary prophylaxis with G-CSF after every cycle of mFOLFIRINOX will follow the institutional practice of the study site (Table 2).

**Table 2:** Treatment regimen mFOLFIRINOX

IMP	Dose [mg/m²]	Route of administration
Oxaliplatin	85	2-hour intravenous infusion
Leucovorin	400	30 min to 2-hour intravenous infusion
Irinotecan	150	90 min intravenous infusion
5-FU	2400	46-hour intravenous infusion

### Dose modifications and prerequisites for the start of a new cycle

A dose modified regimen starting at the first cycle is also allowed at the discretion of the treating physician: irinotecan and oxaliplatin starting dose may be reduced to 80% [1, 5, 6]. Dose adjustments during treatment should be based on the maximum graded toxicity within the previous cycle, graded using the National Cancer Institute Common Toxicity Criteria (version 5.0).

Any toxicity associated with or possibly associated with study drug treatment should be managed with symptomatic treatment and dose modifications. Dose modifications may include dose reduction, dose interruption, and/or dose discontinuation. In case of non recovery after more than 2 weeks delay, stop treatment and refer to sponsor for decision on continuation. If mFOLFIRINOX is interrupted or delayed for > 4 weeks due to toxicity that is suspected to be related to treatment, study treatment should be permanently discontinued. After treatment interruption because of toxicity, the dose of one or more agents of the mFOLFIRINOX regimen may be modified according to maximum graded toxicity within the previous cycle as described below. Dose modifications will be at the discretion of the investigator per medical judgment.

### Prerequisites and surgical procedures after randomization into arm 1

It is requested that the surgical procedures should be performed by the most experienced senior surgeons available in the individual centre. Selection of patients according to the inclusion criteria and eligibility of resectability will be performed in certified tumor boards in each participating centre.

Simultaneous resection is performed when the following criteria are met: (1) No peritoneal carcinomatosis or other organ metastases (except liver metastases) (2) No more than 3 liver metastases (3) Both primary tumor and liver metastases must be considered resectable.

### Judgement of resectability of the primary tumor

Multidisciplinary participation is required for resectability judgement. The resectability of the primary tumor will be judged based on the “Resectability criteria pancreatic adenocarcinoma” of the Dutch Pancreatic Cancer Group (DPCG), 2012, http://www.dpcg.nl. Resectability is determined on preoperative imaging and defined by the DPCG. Resectable disease is defined as no arterial (hepatic artery, superior mesenteric artery, or coeliac trunk) tumor contact and venous (portal vein or superior mesenteric vein) tumor contact of 90 degrees or less. Borderline resectable disease is defined as arterial tumor contact 90 degrees or less and venous contact of 90 to 270 degrees without occlusion (Table 3).

**Table 3:** Resectability criteria for pancreatic adenocarcinoma according to the Dutch Pancreatic Cancer Group, 2012, http://www.dpcg.nl

	SMA superior mesenteric artery	Celiac axis	CHA common hepatic artery	SMV-PV superior mesenteric vein– portal vein
**Resectable** (all four required)	no contact	no contact	no contact	≤ 90° contact
**Borderline resectable** (minimally one required)	≤ 90° contact	≤ 90° contact	≤ 90° contact	90°-270° contact and no occlusion
**Irresectable** (minimally one required)	contact > 90°	contact > 90°	contact > 90°	contact > 270° or occlusion

### Sequence of surgical interventions

1. Resection of one liver metastasis during the first explorative laparotomy or diagnostic laparoscopy is allowed for histological confirmation, preferably an atypical small resection of one peripheral liver metastasis. Macroscopic R0 resection should be achievable for the metastatic lesion. In this situation, the primary tumor will be resected after application of 8 cylces of mFOLFIRINOX chemotherapy in combination with the remaining liver metastases (if more than one but less than 3 liver metastases existed).
2. Explorative laparotomy and resection should be performed within 2–6 weeks after the last mFOLFIRNOX administration according to local institutional practice. Explorative laparoscopy to exclude peritoneal carcinomatosis immediately before laparotomy is optional. All procedures necessary to prepare for surgery, perioperative and postoperative that are standard-of-care, will also follow local institutional practice.
3. Based on the intraoperative findings during explorative laparotomy, the surgeon will evaluate and decide whether resection of the primary tumor in curative intent can be performed. Intraoperative rapid section analyses are obligatory for the pancreatic transection margin to ensure safe and margin-free resection.
4. The resection of the primary pancreatic tumor and the liver metastases can be conducted in the open laparotomy or minimally invasive technique. It is up to the surgeon, to conduct the sequence, first the resection of the metastases and secondly the resection of the pancreatic adenocarcinoma, or vice versa. In this decision, also the role of ablation is relevant which may lead to necrotic parts in the liver which could be infected by a subsequent hepaticojejunostomy.

### Surgical procedures for the liver metastases

1. We have restricted the extent of liver surgery within the trial up to atypical liver resections of a maximum of three metastases.
2. Macroscopic R0 resection or ablation must be achievable for the metastatic lesion(s).
3. A bisegmentectomy of segment II/III can be considered in individual cases.
4. In patients with metastatic lesions located in the center of the liver, requiring major resections, locally ablative techniques (e.g. radiofrequency or techniques available in the respective local study site) can be combined intraoperatively with surgical removal of resectable metastases. We recommend to conduct the ablation sonoguided intraoperatively according to local institutional practice.
5. Alternatively, the ablation can be conducted interventionally (e.g. percutaneous ct-guided) according to local institutional practice. In these cases the liver ablation of selected metastatic lesion(s) can be planned 1–3 weeks after the last mFOLFIRINOX cycle, but within six weeks between the last cycle of chemotherapy and surgery.

### Statistical considerations

#### Sample size calculation

The sample size calculation is driven by the primary endpoint overall survival. Recruitment will be over 5 years and all patients will be followed up for at least 2 additional years, unless the patient dies beforehand, resulting in a maximum follow-up of 7 years. Assuming two-year survival probabilities of 15% in the control arm [1] Fig. 1A mFOLFIRINOX arm) and 30% in the experimental arm a sample size of 109 patients per group will yield a power of 90% at the usual two-sided significance level of 5%. The assumed two-year survival probability of 30% in the experimental treatment arm corresponds to a hazard ratio of 0.635, which is considered clinically relevant. Adjusting conservatively for about 20% dropout (i.e. study discontinuations following randomization) we aim to randomize a total of 272 patients. The sample size calculation was carried out using nQuery (version 8.2.0.0). Assuming that about two thirds of the screened patients will be eligible for randomization we plan to screen about 400 patients.

We will use an adaptive design in METAPANC. Towards the end of the recruitment period, we will conduct a sample size review verifying planning assumptions such as the overall event and dropout rate, i.e. based on non-comparative data (also referred to as blinded sample size re-estimation); the randomization code will be kept confidential and not be used in the sample size review. Furthermore, a futility analysis will be carried out; futility criteria will be based on the overall event rate and recruitment considerations.

#### Efficacy

The primary analysis will follow the intention-to-treat (ITT) principle. The primary outcome survival will be analyzed by a Cox proportional hazards regression. The treatment effect will be reported as hazard ratio with 95% confidence intervals and p-value testing the null hypothesis of no effect (i.e. a hazard ratio of 1). Patients withdrawing from study treatment will be followed up for endpoints. Withdrawal from the study will be dealt with as independent right censoring in the primary analysis unless the patient’s vital status can be obtained at end of study. If withdrawal from study is substantial and differential between the treatment groups, supporting analyses will explore the impact of the independent right censoring assumption by use of shared frailty models for time to death and time to withdrawal from study.

The analyses of the time-to-event outcomes among the secondary endpoints will follow the same lines as the analyses of the primary endpoint. Quality of life (EORTC QLQ-C30, PAN-26, CIPN20, Q-TWIST) and continuous outcomes assessed repeatedly will be analysed using the so-called Mixed Model Repeated Measures (MMRM) approach. Treatment differences will be reported as least square means with 95% confidence intervals.

#### Safety

Standard reporting for adverse events (AEs) and serious adverse events (SAEs). AEs and SAEs will be summarized by frequencies and percentage for each treatment group. AEs will be coded according to MedDRA, analyzed, and presented following ICH E3 Structure and Content of Clinical Study Reports. Events of special interest (e.g. toxicities, post-operative complications) will be analyzed accounting for varying follow-up times and competing events [7].

All details of the statistical analysis including definitions of the analysis sets will be specified in a statistical analysis plan, which will be finalized prior to end of recruitment.

### Assessment of severity/intensity

For the grading of the severity/intensity of an adverse event (AE), the National Cancer Institute Common Toxicity Criteria for Adverse Events (NCI CTCAE) version 5.0 must be used. Medical and scientific judgment should be exercised in deciding whether expedited reporting is appropriate in other situations, such as important medical events that may not be immediately lifethreatening or result in death or hospitalization but may jeopardize the patient or may require an intervention to prevent one of the outcomes listed in the definition above. These events should also usually be considered serious (SAE). All noxious and unintended responses to a medicinal product related to any dose should be considered adverse drug reactions (ADRs). The investigator must report the outcome of AEs and SAEs. All SAEs that have not resolved by the end of treatment visit or discontinuation of IMP treatment, whichever is later, must be followed until the outcome is recovered, recovered with squeal, unchanged/not recovered until death (death due to another cause) or death (due to the SAE). Investigators must report all SAEs immediately within 24 h of knowledge of the event.

### Regulatory, ethical, legal and trial oversight considerations

METAPANC is a phase III clinical trial implemented and reported in accordance with the CTR (EU) 536/2014, ICH-GCP, with applicable Member states regulations, and with the ethical principles laid down in the Declaration of Helsinki. It was submitted, evaluated, and approved via CTIS with Germany as the reporting member state. It is registered under its EU Clinical Trial Number 2023-503558-10-00 in the EU Clinical Trial Register.

### Informed consent

Before enrolment in the clinical trial, the patient will be informed that participation in the clinical trial is voluntary and that he/she may withdraw from the clinical trial at any time without having to give reasons and without penalty or loss of benefits to which the patient is otherwise entitled. The treating physician will provide the patient with information about the treatment methods to be compared and the possible risks involved. At the same time, the nature, significance, implications, expected benefits and potential risks of the clinical trial and alternative treatment will be explained to the patient. During the informed consent discussion, the patient, if applicable, will also be informed about the insurance cover that exists and the insured’s obligations. The patient will be given ample time and opportunity to obtain answers to any open questions. All questions relating to the clinical trial should be answered to the satisfaction of the patient and/or his/her legal representative. In addition, the patient will be given a patient information sheet which contains all the important information in writing.

### Monitoring

In German sites, monitoring is performed by the CRAs of the CTU UMG. Risk-based monitoring will be done according to ICH-GCP E6 and standard operating procedures (SOP) to verify that patients’ rights and wellbeing are protected, reported trial data are accurate, complete and verifiable from source documents and that the trial is conducted in compliance with the currently approved protocol/amendment. All investigators will accept monitoring visits before, during and after the clinical trial to ensure that the trial is conducted, recorded and reported according to the trial protocol, SOPs, ICH-GCP and the applicable regulatory requirements. Prior to the trial, a site initiation visit at each site is conducted in order to train and introduce the investigators and their staff to the trial protocol, essential documents, handling of IMP and related trial specific procedures, ICH-GCP and national/local regulatory requirements. During the trial, the CRA will visit the site regularly depending on the recruitment rate and quality of data. During these on-site visits, the CRA verifies that the trial is conducted according to the trial protocol, trial specific procedures, ICH-GCP and national/local regulatory requirements. The presence of signed informed consents, eligibility of patients, primary endpoint, handling of IMP and documentation/reporting of safety data (e.g. AE/SAE) will be verified by the CRA. The CRA performs also source data verification to ensure that the clinical trial data which are recorded in the source data and eCRFs are complete and accurate. Extent of source data verification and monitor visit frequency will be adapted for individual sites in case of lack of data quality or a high number of protocol violations. All trial specific monitoring procedures, monitoring visit frequency and extent of SDV will be predefined in a trial specific monitoring manual. The investigator must maintain source documents for each patient in the trial, consisting of case and visit notes (hospital or clinic medical records) containing demographic and medical information, laboratory data, electrocardiograms, and the results of any other tests or assessments. All information recorded on eCRFs must be traceable to source documents in the patient’s file. The investigator must also keep the original signed informed consent form (a signed copy is given to the patient). The investigator must give the CRA access to all relevant source documents to confirm their consistency with the eCRF entries.

### Translational research program

The translational research program aims to identify biomarkers for better identification of a potential target population which may benefit from intensified multimodal treatment strategies, to identify molecular biomarkers associated with progression or resistance to study treatment and to identify biomarkers adding to the understanding of PDAC tumor biology. The program will focus on collecting high-quality tissue and liquid biopsy samples, fecal samples and imaging data.

### Exploratory biospecimen analyses

Tissue analyses: samples will be collected and undergo careful pathological quality assessment for subsequent molecular analysis using DNAseq (NGS panel to analyze the mutational status of KRAS, BRCA1/2 and other genes relevant in PDAC pathobiology), RNAseq (to perform signaling pathway analysis, deconvolution of cell lineages and gene expression analysis) and spatial immune profiling (multiplex-Immunofluorescence for tumor and immune markers including tumor markers (cytokeratins, GATA6), fibroblast markers (e.g. alpha-SMA, FAP), immune markers (e.g. CD3, CD4, CD8, Granzyme B) and markers assessing proliferation (KI67) and cell death (e.g. cleaved caspase 3).

Liquid biopsy: collected blood samples will be used to isolate plasma and PBMCs. Quality assessment will be performed, and cell-free DNA and RNA will be isolated for analysis of predictors of response, resistance or toxicity.

Fecal samples: identify biomarkers in the gut microbiome and other biomarkers (e.g. pancreatic enzymes) that are predictive of response to study interventions.

Biological samples drawn during the course of the trial will be collected from the investigator sites and stored and further analyzed by a sponsor assigned central laboratory (West German Biobank and Bridge Institute of experimental Tumor Therapy, West German Cancer Center, University Hospital Essen). Detailed instructions on sample collection, processing, handling and shipment are provided in the laboratory manual (Supplementary Information, Additional file 1).

### Imaging analysis

The development of image-based biomarkers for therapy response prediction and disease outcome presents another exploratory endpoint. For this purpose, baseline and follow up imaging data will be collected for a central review process. Existing and newly developed machine learning (ML) algorithms will be applied to identify PDAC subtypes and predictive therapy response patterns as previously described.

The imaging biomarker translational program includes: secure imaging data transfer and central storage of imaging data, retrospective central image reading and longitudinal imaging data registration, quantitative imaging biomarker extraction (e.g. tumor / metastases, body composition and comorbidities) and ML-based outcome prediction including various clinical endpoints (e.g. therapy response, molecular marker, outcome).

## Discussion

Patients with oligometastatic PDAC and relatively favorable tumor biology may benefit from simultaneous resection of both the primary tumor and liver metastases. This approach could potentially offer a chance for cure or, at least, lead to a survival benefit compared to conventional palliative chemotherapy in selected patients. However, once distant metastases are detected, tumor resection is still not recommended according to German and NCCN guidelines [8, 9], but resection may be considered in selected patients with oligometastases as part of a multimodal treatment approach within the context of clinical trials.

There are no published results from randomized controlled trials on the topic of primary tumor resection in oligometastatic pancreatic cancer. The few systematic reviews consider relatively small case series. The survival prognosis is significantly better in oligometastasis with synchronous resection of the primary tumor and metastases compared to diffuse metastasis [10–12]. Overall, the predominantly retrospective studies provide evidence that overall survival in selected patients with oligometastatic PDAC could be significantly prolonged by primary synchronous resection of both the primary tumor and metastases, compared to patients who are only offered exploration or palliative bypass with chemotherapy [13, 14]. In the multicentre analysis by Tachezy et al., patients who underwent resection of an M1 pancreatic carcinoma showed a significantly longer survival compared to patients who only underwent exploration: 14 months vs. 8 months, but this applies only to the subgroup of patients with pancreatic head carcinoma [15]. These data, however, do not differ significantly from those achieved in the metastatic stage through systemic chemotherapy. In another study, patients with a body/tail pancreatic carcinoma benefited most from a synchronous resection of the primary tumor and liver metastases. A systematic review reports that patients with simultaneous resection of the primary tumor and liver metastases may even have comparable survival rates to patients who underwent resection without distant metastases. These findings are also observed in more recent, small, non-randomized pilot studies, where the tumor marker CA 125 is considered to play a special role as a resection criterion for liver metastases, and a notable degree of patient selection is apparent [16, 17]. The results after neoadjuvant chemotherapy may be more conclusive [11, 18]. In General, data from controlled and randomized studies are missing. In summary, there is evidence suggesting that synchronous resection of the primary tumor and metastases in oligometastatic pancreatic ductal adenocarcinoma, particularly as part of a multimodal treatment strategy, leads to better overall survival outcomes compared to diffuse metastatic pancreatic cancer. How these results compare to a state-of-the-art systemic therapy without resection must be determined by prospective, randomized studies. Until then, resection of the primary tumor and metastases is not yet a clinical standard, even in oligometastatic cases [19].

In the palliative setting, chemotherapy regimens such as FOLFIRINOX or gemcitabine/nab-paclitaxel have recently been established, demonstrating a significant improvement in overall survival (OS), with medians of 11 and 8.5 months, respectively, compared to 7 or 6.7 months with gemcitabine monotherapy [1, 20].

Multimodal treatment strategies, including local therapies, are recommended to enhance disease control and clinical outcomes in these patients. Distinct clinical courses of oligo- and polymetastasis are observed in tumors of different origins, and a comprehensive approach involving multiple entities is required to identify common underlying mechanisms. In pancreatic cancer, the concept of oligometastasis has not yet been established in clinical practice. Furthermore, it is currently unclear whether true oligometastatic disease exists in pancreatic cancer, as it does in colorectal cancer at known stages. The challenge right now is how to select patients who potentially profite from multimodal surgery treatment. Thus finding a strategy to select patient groups who most likely benefit from additional surgery is urgently needed. In this context the METAPANC trial aims to evaluate the effectiveness of multimodal therapy in patients with the clinical presentation of oligometastasis in pancreatic cancer. The combination of highly effective polychemotherapy (mFOLFIRINOX) followed by complete tumor resection is intended to support the potential for curative treatment in these patients — a group that has previously been considered exclusively palliative. The primary objective of this randomized phase III study is to demonstrate the efficacy of this therapeutic concept in terms of overall survival.

METAPANC is currently the only prospective, randomized, controlled, multicentre, phase III trial for curatively intended surgical therapy of oligometastatic pancreatic cancer in Western Europe and the Anglo-American world. In the Asian region, the CSPAC-1 study is currently being conducted as another prospective randomized study in China [4]. As a Phase II study on multimodal therapy for hepatically metastasized pancreatic adenocarcinoma, the HOLIPANC study investigates, in a single-arm study design, the effectiveness and quality of life of a neoadjuvant chemotherapy regimen consisting of liposomal irinotecan combined with oxaliplatin and 5-fluorouracil. The study also evaluates the significance of a curatively intended resection of the primary tumor and oligometastases (up to a maximum of 5 liver metastases).

The multimodal surgical therapy of patients with oligometastatic pancreatic cancer could significantly extend the survival of this patient group within the framework of the METAPANC trial. In order to recommend this treatment regimen outside of clinical trials, data from randomized controlled studies are needed first. An exploratory translational research program is implemented to identify patient groups that could benefit from multimodal surgical therapy. In Germany 26 institutions are currently planned as participating centres (Supplementary Information, Additional file 2). Additionally, international high-volume centres from the Netherlands, Finland, Sweden, and Norway are in preparation. The recruitment of patients has already started. The METAPANC trial is expected to run for at least seven years.

## Electronic supplementary material

Below is the link to the electronic supplementary material.

Supplementary Material 1: Additional file 1. Laboratory manual

Supplementary Material 2: Additional file 2. Table 1: German METAPANC centres

Supplementary Material 3

## Data Availability

No datasets were generated or analysed during the current study.

## Abbreviations

5-FU: 5-fluorouracil
ADR: Adverse drug reaction
AE: Adverse event
ACT, ATC: Anatomical Therapeutic Chemical (Classification System)
alpha-SMA: Alpha Smooth muscle actin
AWMF: Association of the Scientific Medical Societies in Germany (Arbeitsgemeinschaft der Wissenschaftlichen Medizinischen Fachgesellschaften)
BRCA1/2: Breast Cancer Gene 1/2
CA 125: Cancer antigen 125
CA19-9: Carbohydrate Antigen 19 − 9
CD3: Cluster of Differentiation 3
CD4: Cluster of Differentiation 4
CD8: Cluster of Differentiation 8
CHA: Common hepatic artery
CRA: Clinical research associate
CR: Complete remission
CSPAC: Chinese Study Group for Pancreatic Cancer
CTR: Clinical Trials Regulation
CT: Computer Tomography
CTIS: Clinical Trials Information System
CTU UMG: Clinical trials unit University Medicine Göttingen
DPCG: Dutch Pancreatic Cancer Group
DNAseq: DNA sequencing
ECOG: Eastern Cooperative Oncology Group (ECOG Performance Status Scale)
eCRF: Electronic Case Report Form
EORTC: European Organisation For Research And Treatment Of Cancer
EORTC QLQ-C30: EORTC Core Quality of Life questionnaire
EORTC QLQ-PAN26: EORTC health-related quality of life for people with pancreatic ductal adenocarcinoma
FAP: Fibroblast activation protein
GATA6: GATA binding protein 6
GCP: Good Clinical Practice
G-CSF: Granulocyte colony stimulating factor
ICH: International Council for Harmonisation of Technical Requirements for Pharmaceuticals for Human Use
IMP: Investigational Medicinal Product
ITT: Intention-to-treat
KI67: Marker of proliferation Kiel 67
KRAS gene: Kirsten rat sarcoma virus proto-oncogene
MedDRA: Medical Dictionary for Regulatory Activities
mFOLFIRINOX: Modified FOLFIRINOX
ML: Machine Learning
MRI: Magnetic Resonance Imaging
NCCN: National Comprehensive Cancer Network
NCI CTCAE: National Cancer Institute Common Terminology Criteria for Adverse Events
NGS: Next generation sequencing
NYHA: New York Heart Association (NYHA Functional Classification)
OS: Overall survival
PBMC: Peripheral Blood Mononuclear Cell
PD: Progressive disease
PDAC: Pancreatic ductal adenocarcinoma
PFS: Progression-free survival
PR: Partial response
PV: Portal vein
Q-TWIST: Quality-adjusted time without symptoms or toxicity
RECIST v1.1: Response Evaluation Criteria in Solid Tumors version 1.1
RNAseq: RNA sequencing
SAE: Severe adverse event
SD: Stable disease
SDV: Source Data Verification
SMA: Superior mesenteric artery
SmPC: Summary of product characteristics
SMV: Superior mesenteric vein
SOP: Standard operating procedure
WBC: White Blood Cell
