# Supplementary material for: Study protocol of the METAPANC trial - intensified treatment in patients with local operable but oligometastatic pancreatic cancer - multimodal surgical treatment versus chemotherapy alone: a randomized controlled trial
Source: BMC Cancer. 2025 Feb 6;25:208. doi: 10.1186/s12885-025-13573-7 (PMC11800597; doi:10.1186/s12885-025-13573-7)
Supplement: Supplementary file 1 — Supplementary Material 1: Additional file 1. Laboratory manual [file 12885_2025_13573_MOESM1_ESM.pdf]

LABORATORY MANUAL

Intensified treatment in patients with oligometastatic pancreatic cancer - multimodal surgical treatment versus systemic chemotherapy alone: a randomized controlled trial

(AIO-PAK-0219 METAPANC)

Protocol Number: FOMA-ID 2020-01069
EU CT No.: 2023-503558-10-00
Version: 1.1.2
Date: 20.06.2024

Lead Coordinating Clinical Trial Investigator: Prof. Dr. med. Michael Ghadimi

Lead Coordinating Translational Program Investigator: Prof. Dr. med. Jens Siveke

Document	Name / Sign	Date
Author:	Dr. Sven-Thorsten Liffers	20.06.2024
Approved by:	Prof. Dr. Jens Siveke	
Version/changes:	V1.1.2	20.06.2024

Index of contents

1. List of Abbreviations 3
2. List of contacts 4
3. About this manual 5
4. Laboratory assessments, material and procedures of samples 6
4.1. Local lab sample processing 6
4.1.1 Tumor Biopsy Sample 6
4.1.2 Serum samples for biomarker analysis (Exploratory studies) 8
4.1.3 Plasma samples for biomarker analysis (Exploratory studies) 9
4.1.4 PBMC samples for biomarker analysis (Exploratory studies) 13
4.1.5 Fecal samples for biomarkers (Exploratory studies) 14
5. Imaging assessment 13
6. Questions, Resupply and Samples' Shipment 15

1. List of Abbreviations

ACK	- Ammonium-Chloride-Potassium
C01_D01	- Cycle 1 Day 1
EDTA	- Ethylenediaminetetraacetic acid
EOT	- End of Treatment
FFPE	- Formalin-fixed Paraffin-embedded
PBMC	- Peripheral blood mononuclear cell
RBC	- Red blood cells (Erythrocytes)
PBS	- Phosphate-Buffered Saline

2. List of contacts

Function	Contact
**Coordinating Principal Investigator**	**Prof. Dr. med. Michael Ghadimi**<br>Universitätsmedizin Göttingen<br>Klinik für Allgemein-, Viszeral- und Kinderchirurgie<br>Georg-August-Universität<br>Robert-Koch-Straße 40<br>37075 Göttingen / Germany<br>**Phone:** +49 (0) 551 3967811<br>**E-Mail:** chirurgie.sekretariat@med.uni-goettingen.de
**Coordinating Translational Program Principal Investigator**	**Prof. Dr. med. Jens Siveke**<br>Bridge Institute of Experimental Tumor Therapy<br>West German Cancer Center<br>University Hospital Essen<br>Hufelandstr. 55, 45147 Essen / Germany<br>**Phone:** +49 (0) 201 723 3704<br>**E-Mail:** jens.siveke@uk-essen.de
**Coordinator Translational Sample Collection**	**Dr. Sven-Thorsten Liffers**<br>Bridge Institute of Experimental Tumor Therapy<br>West German Cancer Center<br>University Hospital Essen<br>Hufelandstr. 55, 45147 Essen / Germany<br>**Phone:** +49 (0) 201 723 82462<br>**E-Mail:** sven-thorsten.liffers@uk-essen.de
**Sponsor**	**University Medical Center Göttingen (UMG)**<br>**Dept.: Clinical Trial Unit (Studienzentrum UMG)**<br>**represented by the Head of Clinical trials unit UMG**<br>Von-Bar-Str.2/4<br>37075 Göttingen / Germany<br>**Phone:** +49 (0) 551 39 60812<br>**E-Mail:** sz-umg.sponsor-qm@med.uni-goettingen.de
**Project Manager (PM)**	**Dr. Ralph Telgmann**<br>University Medical Center Göttingen (UMG)<br>Clinical Trial Unit (Studienzentrum UMG)<br>Von-Bar-Str.2/4<br>37075 Göttingen / Germany<br>**Phone:** +49 (0) 551 39 60821<br>**E-Mail:** ralph.telgmann@med.uni-goettingen.de
**Central Biobank Essen**	**Westdeutsche Biobank Essen**<br>Universitaetsklinikum Essen<br>**Head Biobank:** Dr. Katharina Jockers<br>Hufelandstraße 55<br>45147 Essen / Germany<br>**Phone:** +49 (0) 201 723 1667<br>**E-Mail:** katharina.jockers@uk-essen.de

3. About this manual

This manual describes in detail how to collect and process the research samples (i.d. tumor biopsies, serum, plasma and PBMC samples) obtained in the METAPANC trial. The manual shall ensure a consistent and high standard of quality for the intended biomarker research.

This manual provides staff with the general overview of laboratory assessments that are required during this trial, a list of parameters, the lab material needed and provided for handling of samples. The document highlights for the site and laboratory staff the way in which each sample is collected, prepared/processed, handled, labelled and transported. The handling of samples ultimately has a direct impact on the overall success of the study. Incorrect handling of clinical trial samples can result in false or misleading measurements and can negatively affect the results of the trial.

The identity, quality and integrity of the sample for its given lifetime must be assured by all parties involved in the sample process.

All tumor tissue, blood-based and fecal samples obtained for the described research studies are biobanked in the **central lab in Essen**, Germany.

4. Laboratory assessments, material and procedures of samples

Please make sure that each patient has provided a written informed consent to participate in the Translational Research Program.

Please make sure that all lab results are documented appropriately on lab reports in the subject's source data. All lab reports need to be checked by an investigator and clinical significance of values needs to be assessed, commented and initialed with date. Please ensure that subjects do not meet any of the exclusion criteria when performing the screening assessment.

Please, carefully observe the time of sample collection and temperatures for sample shipment, which are provided in detail in the following sections.

4.1. Local lab sample processing

4.1.1 Tumor Biopsy Sample <u>(Exploratory Research Studies)</u>

Sample	Assessment planned	Time of sampling
biopsy cores	Tumor Biopsy	▪ Screening<br>▪ Resection (Arm A)<br>▪ time of progression (optional)

Baseline tumor tissue samples will be collected from all patients, preferably by means of a biopsy performed at study entry. If a biopsy is not deemed feasible by the investigator, archival tumor tissue from resected tumor (primary and metastases) or biopsies (primary and metastases) may be submitted after sponsor approval has been obtained.

Tumor biopsy samples at resection of tumors (in Arm A "surgery") will be collected from all patients at date of resection.

Additionally, a recommended new biopsy should be requested at EOT from participants whose disease have progressed if deemed clinically feasibly by the investigator.

<u>Preparation/processing of tumor tissue samples</u>

Make sure that only one biopsy core is present per FFPE-block. Request the blocks from your pathology department after initial tumor evaluation. In case it is impossible to get the block, you can alternatively request at least 16 slides unstained, freshly cut, serial sections.

If more than two cores are taken up to, two cores should be fresh frozen. Therefore, place each core seperately in one of the provided cryo vial (FluidX Cryo - Tissue sample tube 68-4000-01) and immediately snap freeze in liquid nitrogen and store in a long-term storage freezer (-130°C or below) until requested for central shipment and analysis. Please, enter the relevant information (i.e. patient_id, date of birth, collection_label, collection_time, start time

(Processing), sample type, biopsies/vial, rack_id, position; tube_id) in the Fresh_Frozen_Tissue sheet of the provided Excel document.

In case the samples can be stored only at -80°C samples should be shipped within 12 months to UME Essen. For shipping details on dry ice refer to section 5

4.1.2 Serum samples for biomarker analysis (Exploratory Research Studies)

Please make sure, that the patient has consented to the biomaterial collection before taking samples.

Sample needed:	Assessment planned:	Time of sampling
9.0 mL serum sample	Biomarker	▪ Cycle 1-Day 1 (C01D01)<br>▪ Cycle 2-Day 1 (C02D01)<br>▪ Response Assessment I (RA01)<br>▪ Response Assessment II (RA02)<br>▪ EOT (30 days ± 5 days after last dose)

Material needed	Company	Order-Number
S-Monovette Serum-Gel	e.g. Sarstedt	02.1388
Plasma tubes – 500 µL	FluidX	68-0701-10

Instructions

- collect 1 x 9 mL serum sample
- allow blood to clot for 15 – 30 min, tube standing upright at room temperature
- centrifuge at room temperature for 10 min at 2000 xg
- use pipette to transfer 500 µL serum (supernatant) aliquots into clean and pre-chilled provided plasma tubes
- record the following information in the provided excel sheet:

patient_id
date of birth
collection_label (e.g. C01D01)
collection date
collection time
start time (Processing)
sample type - Serum
volume [µL]
rack_id
position
tube_id

- Immediately store at -80°C (if not available, store at -20°C)

➲ Ship on dry ice upon request (for shipping details refer to section 5)

4.1.3 Plasma and PBMC isolation for biomarker analysis (Exploratory Research Studies)

Please make sure, the patient has consented to the biomaterial collection before taking samples.

Flow Chart: Preparation of Plasma and PBMC from Whole Blood Samples

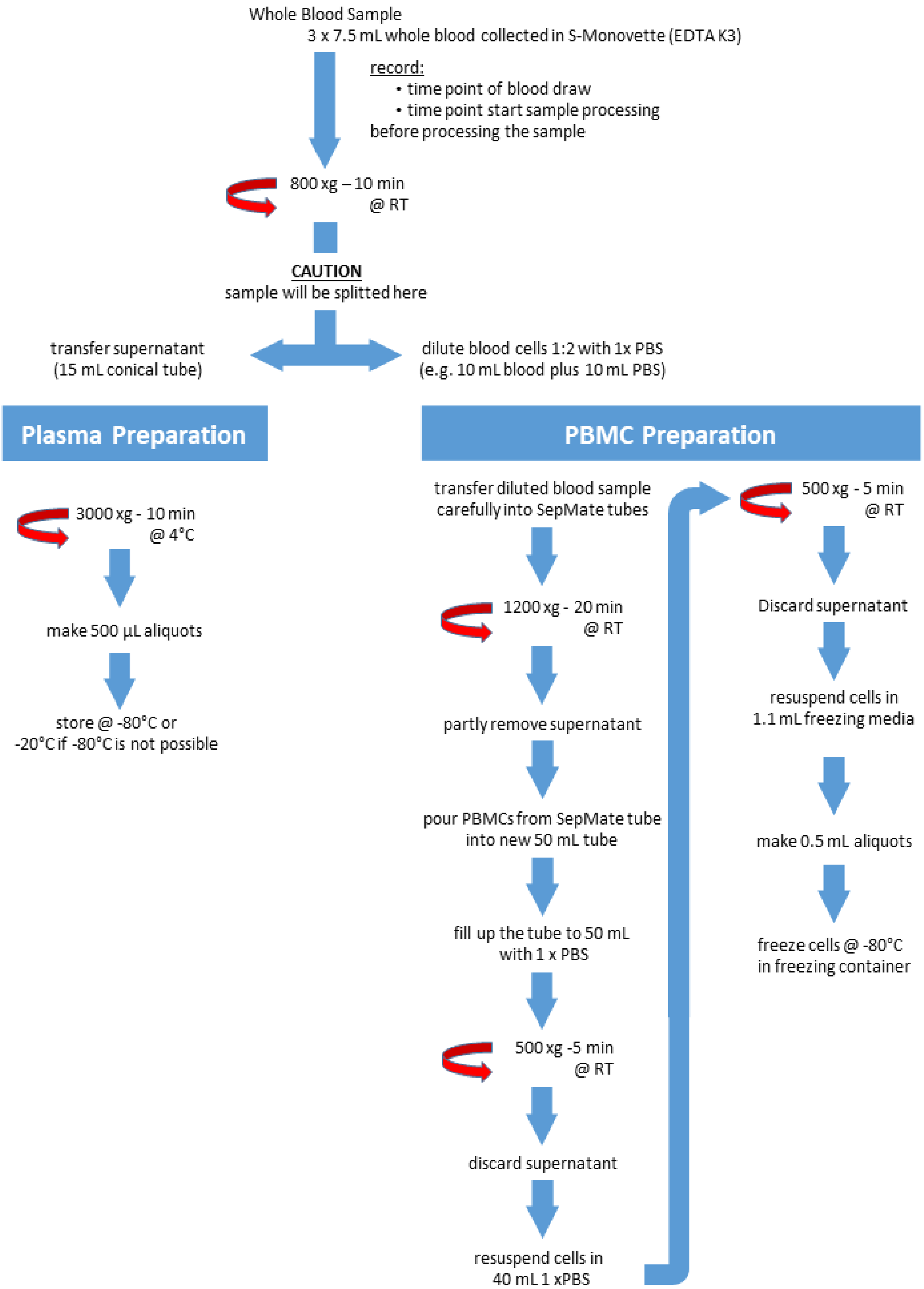

Figure 1: Overview of Plasma and PBMC preparation from of 3x 7.5 mL EDTA whole blood samples.

Sample needed:	Assessment planned:	Time of sampling
3x 7.5 mL EDTA sample	Biomarker	▪ Cycle 1-Day 1 (C01D01)<br>▪ Cycle 2-Day 1 (C02D01)<br>▪ Response Assessment I (RA01)<br>▪ Response Assessment II (RA02)<br>▪ EOT (30 days ± 5 days after last dose)

Material needed	Company	Order-Number
S-Monovette (EDTA K3)	e.g. Sarstedt	01.1605.001
Conical tube – 50 ml	e.g. Sarstedt	62.547.254
Conical tube – 15 ml	e.g. Sarstedt	62.554.502
Plasma tubes – 500 µL	FluidX	68-0701-10
Cryo tubes – 1.9 mL	FluidX	65-7641

Instructions

- collect 3 x 7.5 mL EDTA samples

! Sample must be processed within less than 4h after blood drawing !

- record date/time of blood collection as well as date/time of starting sample processing
- centrifuge SepMate-50 tubes at 100 xg for 5 min to make sure that the Pancoll is in the lower compartment of the tube
- pool whole blood samples in a 50 mL conical tube
- separate plasma from blood cells by centrifugation at 800 xg for 10 min at room temperature
- collect supernatant (plasma) up to 5 mm above the leucocyte phase and transfer into a new 15 mL conical tube and store on ice
- dilute the remaining whole blood cells with 1x PBS in a 1:2 ratio

e.g. 10 mL blood plus 10 mL PBS

Note: minimal volume is 15 ml; fill up with PBS if not enough blood present

A. *Plasma Preparation*

- centrifuge the 15 mL tube (plasma sample) at 3000 xg for 10 min at 4°C
- transfer 500 µL aliquots of the supernatant into pre-chilled plasma tubes without touching the pellet
- record the following information in the provided excel sheet:

  patient_id
  date of birth
  collection_label (e.g. C01D01)
  collection date
  collection time
  start time (Processing)
  sample type - Plasma
  volume [µL]
  rack_id
  position
  tube_id

Immediately store at -80°C,if -80°C is not feasible store at -20°C, at study site

➲ For shipping details on dry ice refer to section 5

B. *PBMC Preparation*

transfer up to 30 mL of the diluted blood sample into the provided SepMate tubes. Hold the pipette against the tube wall and let the sample slowly run into the tube.
!!! Do not put the sample directly on top of the membrane !!!

- separate the PBMCs by centrifugation at 1200 xg for 20 min at room temperature with brake on
- Remove the supernatant from top until 15 mL are left above the membrane in the SepMate tube (e.g. if you applied 30 mL than remove 15 mL)
- Gently scrape off the PBMCs from the wall with a pasteur pipette
  Pour the remaining 15 mL quickly into a clean 50 mL tube by inverting the SepMate tube.
  !!! Do not hold the SepMate tube inverted for longer than 2 sec. !!!
- fill up the 50 mL conical tube with PBS
- centrifuge at 500 xg for 5 min at room temperature
- aspirate the supernatant without disturbing the pellet
- add 40 mL PBS and resuspend PBMC by inverting the tube
- centrifuge at 500 xg for 5 min at room temperature
- If the pellet shows a contamination of red blood cells, remove the red blood cells via step C “Removal of red blood cells” before counting and freezing. If the pellet is clean, skip step C

C. Removal of red blood cells

- resuspend the pellet in 1mL PBS
- add 9 mL ACK Buffer
- incubate for 10 min at RT
- fill up to 50 mL with PBS
- centrifuge at 500 xg for 5 min at RT
- if the pellet is still contaminated, repeat until the pellet does not show any red blood cell contamination, Otherwise continue to step D.

D. Cell freezing

aspirate the supernatant and resuspend pellet in 1.1 mL freezing media

- Transfer aliquots of 500 µL into provided cryovials (1.9 mL)
- transfer cryo tubes into a freezing container and freeze cells at -80°C overnight
- record the following information in the provided excel sheet:

  patient_id
  date of birth
  collection_label (e.g. C01D01)
  collection date
  collection time
  start time (Processing)
  sampletype
  volume [µL]
  Rack ID
  Position
  Tube ID

- next day remove PBMC tubes from the freezing container and store the tubes at ≤ -130 °C. if only storage at -80°C is possible, samples should be shipped within 12 months to central lab at UME.

➲ Ship on dry ice upon request (for shipping details refer to section 5)

Note: In case the centrifugation cannot carried out at 4°C (preferred!), the whole sample processing can be done at room temperature as long as the sample preparation is done under site specific conditions.

4.1.4 Cell-Free DNA samples for biomarker analysis (Exploratory Research Studies)

Please make sure, the patient has consented to the additional biomaterial collection before taking samples.

☞ **IMPORTANT**

samples should be sent on Monday – Wednesday for stability reason if possible

Sample needed:	Assessment planned:	Time of sampling
1x 10 mL Cell-Free DNA BCT®	Biomarker	▪ Cycle 1-Day 1 (C01D01)<br>▪ Cycle 2-Day 1 (C02D01)<br>▪ Response Assessment I (RA01)<br>▪ Response Assessment II (RA02)<br>▪ EOT (30 days ± 5 days after last dose)

Instructions	Visual Representation
• Collect 1x 10ml Cell-Free DNA BCT® sample<br>• After removal from adapter immediately, **mix gently by inversion the tube 8 to 10 times. Do not shake the sample!**<br><br>Note: Inadequate or delayed mixing may result in clotted blood, which leads to incorrect analytical results and poor product performance.	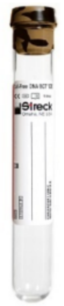

• label the tube with the following information:

on the side: Patient ID
Date
Time of sampling (e.g. C01D01)

➲ Ship ambient at collection day (for shipping details refer to section 5)

4.1.5 Fecal samples for biomarkers (Exploratory Research Studies)

Please make sure, the patient has consented to the additional biomaterial collection before taking samples.

Sample needed:	Assessment planned:	Time of sampling
Fecal sample	Biomarker	▪ Cycle 1-Day 1 (C01D01)<br>▪ Cycle 2-Day 1 (C02D01)<br>▪ Response Assessment I (RA01)<br>▪ Response Assessment II (RA02)<br>▪ EOT (30 days ± 5 days after last dose)

Instructions	Visual Representation
• Instruct patient on handling<br>• Hand out fecal sample kit to patient after filling the accompanying form.<br>• Only the date of sample collection must be entered by the patient	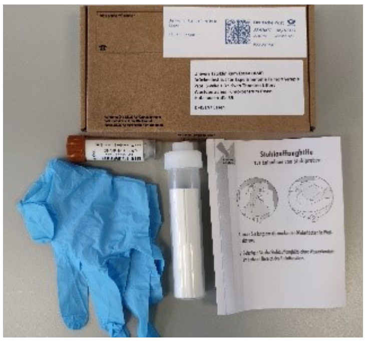
• Patient has to collect stool sample in this collection tube and ship with prepaid card box	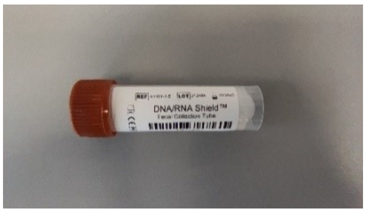

➲ To be shipped ambient by the patient at the day of collection

(address and boxes for shipping are provided with the fecal sample kit)

5. Imaging assesment

In METAPANC, central storage of imaging data is performed to identify and evaluate biomarkers and patterns for identification of a potential target population which may benefit from the intensified multimodal treatment strategy.

UME provides a web-browser based upload service (1) that takes DICOM studies and uploads them to a staging system in the UME IT-infrastructure. For compliance with data protection rules, all patient identifiers will be removed from the DICOM instances before upload according to the Application Level Confidentiality Profiles (2) of the DICOM specification.

The de-identification takes place within the user's webbrowser using a client side DICOM de-identification library created by UME and released as open source (3). In addition, all DICOM instances having the BurnedInAnnotation attribute (0028,0301) set to 'YES' or the Modality attribute (0008,0060) set to 'SC' will be excluded from the upload process because patient identifying information could be contained in burned-in annotations. This process guarantees the complete de-identification of the DICOM data already within the user's web-browser before the upload to UME servers.

Images will be uploaded on the following link (website example as below):

https://dicomupload.uk-essen.de/METAPANC

Please upload using the Patient-ID for the correct pseudonymization process.

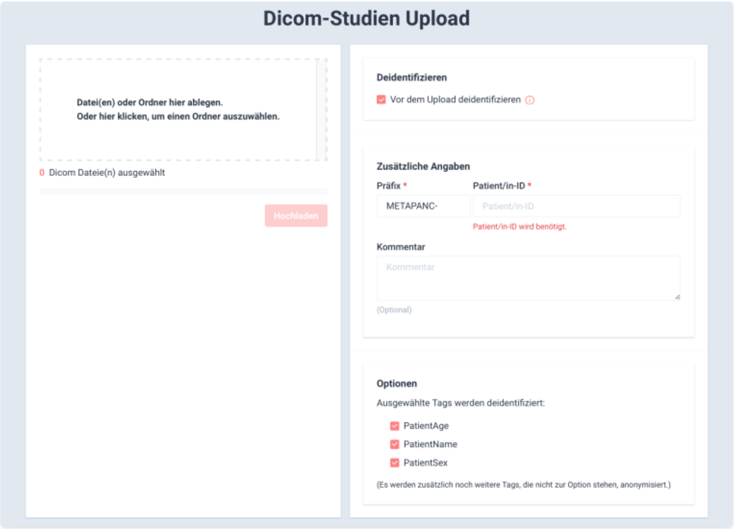

References

[1] https://dicomupload.uk-essen.de

[2] https://dicom.nema.org/medical/dicom/current/output/html/part15.html#table_E.1-1

[3] https://github.com/UMEssen/dicom-deidentifier-ts

6. Questions, Resupply and Samples' Shipment

In case of any questions, resupply and arranging shipments, please feel free to contact:

Email: **sven-thorsten.liffers@uk-essen.de** (preferred)

Telephone: **+49 (0)201-723 82462**

Key points for shipments on dry ice:

- ✓ For all shipments on dry ice contact Dr. Liffers to initiate the sample pick up. We will provide then the shipment details.

- ✓ Prior to shipment, please check that all samples are labeled with the appropriate identifiers (site, patient and visit number etc.) and all the required information on the sample submission form have been completed.

- ✓ Make sure each patient's sample and the completed sample submission form for that patient are packed together by shipping condition.

- ✓ Tumor tissue samples and shipments at room temperature (i.e. 10ml Cell-Free DNA BCT and Fecal samples) are sent by regular mail to:

  Universitätsklinikum Essen (AöR)
  Brückeninstitut für Experimentelle Tumortherapie
  Prof. Dr. Jens Siveke / Dr. Sven-Thorsten Liffers
  Westdeutsches Tumorzentrum Essen
  Hufelandstraße 55
  D-45147 Essen
