# Supplementary material for: Study protocol of the METAPANC trial - intensified treatment in patients with local operable but oligometastatic pancreatic cancer - multimodal surgical treatment versus chemotherapy alone: a randomized controlled trial
Source: BMC Cancer. 2025 Feb 6;25:208. doi: 10.1186/s12885-025-13573-7 (PMC11800597; doi:10.1186/s12885-025-13573-7)
Supplement: Supplementary file 2 — Supplementary Material 2: Additional file 2. Table 1: German METAPANC centres [file 12885_2025_13573_MOESM2_ESM.docx]

| **Center number** | **Center** | **Institution** |
| --- | --- | --- |
| 1 | Goettingen | University Medical Center Göttingen |
| 2 | Essen | University Hospital Essen |
| 3 | Berlin | Charité - University Medicine Berlin |
| 4 | Bochum | Catholic Hospital Bochum gGmbH  St. Josef-Hospital Bochum |
| 5 | Hamburg-Eppendorf (UKE) | University Medical Center Hamburg-Eppendorf |
| 6 | Hanover | Medical University of Hanover |
| 7 | Mannheim | University of Heidelberg  Faculty of Medicine Mannheim |
| 8 | Muenster | University Hospital Muenster |
| 9 | Munich (LMU) | LMU Clinic Munich |
| 10 | Munich (MRI) | TUM Universitätsklinikum Rechts der Isar Hospital Technical University München |
| 11 | Regensburg | Hospital Barmherzige Brüder Regensburg |
| 12 | Mainz | University Medical Center Johannes Gutenberg-University Mainz |
| 13 | Lübeck | University Medical Center Schleswig-Holstein Campus Lübeck |
| 14 | Freiburg | University Hospital Freiburg |
| 15 | Cologne | University Hospital Cologne |
| 16 | Ulm | University Hospital Ulm |
| 17 | Heidelberg | University Hospital Heidelberg |
| 18 | Würzburg | University Hospital Würzburg |
| 19 | Aachen | University Hospital RWTH Aachen |
| 20 | Halle | University Hospital Halle (Saale) |
| 21 | Frankfurt | Hospital Nordwest GmbH |
| 22 | Hamburg (Asklepios) Altona | Asklepios Clinics Hamburg GmbH Asklepios Tumorcenter Hamburg  Asklepios Clinic Altona |
| 23 | Hamburg (Asklepios) Barmbek | Asklepios Clinics Hamburg GmbH Asklepios Tumorcenter Hamburg MVZ Oncology Barmbek |
| 24 | Dresden | Technical University Dresden  Faculty of Medicine Carl Gustav Carus |
| 25 | Erlangen | University Hospital Erlangen |
| 26 | Gießen | University Hospital Gießen |

**Table 4** Participating German METAPANC sites
